# Prevalence of Oropharyngeal Dysphagia and Its Value as a Prognostic Factor in Community-Acquired Pneumonia: A Prospective Case-Control Study

**DOI:** 10.7759/cureus.55310

**Published:** 2024-03-01

**Authors:** Amando Márquez-Sixto, Javier Navarro-Esteva, Lucía Yomara Batista-Guerra, David Simón-Bautista, Felipe Rodríguez-de Castro

**Affiliations:** 1 Pulmonary Medicine, Hospital Universitario de Gran Canaria Doctor Negrín, Las Palmas de Gran Canaria, ESP; 2 Physical Medicine and Rehabilitation, Complejo Hospitalario Universitario Insular-Materno Infantil, Las Palmas de Gran Canaria, ESP

**Keywords:** mortality, v-vst, eat-10, oropharyngeal dysphagia, community-acquired pneumonia

## Abstract

Background: Although oropharyngeal dysphagia (OD) is a common finding in patients with community-acquired pneumonia (CAP), specific recommendations are not provided in the current clinical guidelines.

Objectives: To estimate the prevalence of OD and its associated factors among patients hospitalized for CAP and to assess one-year outcomes according to the presence or absence of OD.

Methods: We studied 226 patients hospitalized for CAP and 226 patients hospitalized for respiratory conditions other than CAP. We screened the risk of OD using the Eating Assessment Tool-10 (EAT-10), followed by the volume-viscosity swallow test (V-VST).

Results: A total of 122 (53.9%) patients with CAP had confirmed OD compared with 44 (19.4%) patients without CAP. Patients with CAP and OD were older (p < 0.001; 1.02-1.07) and had less familial/institutional support (p = 0.036; 0.12-0.91) compared to patients with CAP and no OD. OD was more prevalent as the CURB-65 score increased (p < 0.001). Patients with OD spent more time in the hospital (14.5 vs. 11.0 days; p = 0.038) and required more visits to the emergency room (ER). Twenty (16.4%) patients with CAP and OD died after discharge vs. one (0.8%) patient with CAP and no OD (p < 0.001; CI = 2.24-42.60).

Conclusions: The prevalence of OD in hospitalized patients with CAP is higher than in patients hospitalized for other respiratory diagnoses. Advanced age, lower familial/institutional support, and increased CAP severity are associated with OD. Patients with CAP and OD are more frequent ER visitors after discharge and have a higher mortality. In patients with CAP and OD, aspiration pneumonia is likely underestimated.

## Introduction

Community-acquired pneumonia (CAP) is the leading cause of death of infectious origin in Europe and is a frequent cause of hospitalization. The annual incidence of CAP in adults in Western countries is approximately one per 1000 person-years but is significantly higher in older individuals [1-3]. Of the patients diagnosed with CAP, 30-40% require hospital admission, and it is estimated that one in five patients will be readmitted within 30 days of discharge. The economic burden associated with CAP could be mitigated by reducing the associated risk factors such as polypharmacy in the elderly (e.g., chronic intake of antacids, inhaled/oral corticosteroids, or hypnotics/sedatives) and improving the rates of pneumococcal vaccination [4-6]. Current clinical guidelines for CAP do not address risk factors, including oropharyngeal dysphagia (OD) [1-6]. This study aims to investigate whether OD is more prevalent among patients hospitalized for CAP than among those admitted for a different respiratory diagnosis, to describe the association between OD and the severity of CAP, and to assess whether CAP patients with OD have an increased risk of hospital readmission and mortality compared to CAP patients without OD.

## Materials and methods

Study design/study population

We performed a prospective case-control analysis of all consecutive adult patients (aged ≥ 18 years) with CAP admitted to the respiratory ward of the University Hospital of Gran Canaria Dr. Negrín (a tertiary-care, referral center) between February 2019 and February 2021. The control group comprised patients admitted to the ward within the same period for acute respiratory conditions other than pneumonia. Informed consent was obtained before inclusion in the study. Exclusion criteria were age < 18 years, COVID-19 infection, hospital-acquired pneumonia, active lung cancer, and immunosuppression (including the use of systemic corticosteroids in the previous 90 days). Patients who presented with a low level of consciousness on admission (Glasgow Coma Scale score < 9), did not provide informed consent, or died during admission were also excluded. Several comorbidities were registered for comparison, including chronic obstructive pulmonary disease (COPD), neurological diseases (cerebrovascular accident, Parkinson's disease, dementia), cardiovascular diseases (high blood pressure, coronary artery disease, congestive heart failure), diabetes, chronic renal failure, gastrointestinal disorders (gastroesophageal reflux, chronic liver disease), drugs (benzodiazepines, antidepressants, antipsychotics), and intake of intoxicants, such as tobacco smoking and daily alcohol intake. We also evaluated the presence of OD according to the severity of CAP and the length of hospital stay. Subsequent visits to the emergency room (ER), pneumonia recurrence, and mortality within one year of hospital discharge were assessed by reviewing the patients' electronic charts. Variables including age, Barthel index, institutional or family support, COPD, neurological diseases, and use of benzodiazepines were included in the multivariate analysis. We chose these variables because they are associated with CAP as well as with OD [4-6].

Interventions

An experienced nurse screened for OD in all patients within the first two days of admission or as soon as the patient was capable of cooperating. Initial screening was performed using the Eating Assessment Tool-10 (EAT-10). This is a validated, self-administered questionnaire that uses easily understandable language [7]. It has been validated in Spanish and is considered abnormal when the score is > 3 [8]. In such cases, the volume-viscosity swallow test (V-VST) was subsequently performed as a confirmatory test. V-VST is a safe, simple, and easily applicable method, both at the office and bedside. It employs three different viscosities (pudding, nectar, and liquid) and three swallow volumes (5, 10, and 20 ml) for each viscosity. It starts with nectar viscosity and increasing bolus volume, then liquid and finally pudding viscosity in a progression of increasing difficulty to protect patients from aspiration. The V-VST helps to assess signs of impaired swallowing safety, such as cough, changes in voice quality, or pulse oxygen saturation (≥3% decrease suggests aspiration). It also helps to investigate signs of impaired swallowing efficacy, such as poor labial seal, multiple swallows per bolus, and oropharyngeal residue. To optimize the reliability of the findings, the V-VST was performed after the patient's clinical improvement and immediately before discharge. Both EAT-10 and V-VST have been compared to instrumental methods of diagnosis, showing adequate diagnostic reliability, especially in the case of V-VST [9-11]. Dietary modifications and oral hygiene habits were recommended to any patient with OD at hospital discharge, and follow-up phone calls were conducted in the CAP cohort to assess vital status and compliance with the recommendations. This study protocol was reviewed and approved by the Local Committee of Ethics and Investigation With Drugs of Las Palmas (Comité Ético de Investigación con Medicamentos Provincial Las Palmas CEI/CEIm) (reference number # 2018-007-1).

Statistical analysis

The mean, standard deviation, median, and 25th and 75th percentiles were calculated for the quantitative variables. The Kolmogorov-Smirnov or Shapiro-Wilk tests were used to verify the normality of the data (if the sample size was > 50, the Kolmogorov-Smirnov test was applied; otherwise, the Shapiro-Wilk test was used). The frequencies and percentages of qualitative variables were calculated. The dependence of qualitative variables was analyzed using Fisher's exact test and ordinal variables were analyzed using the linear trend test. Student's T test or Mann-Whitney U test was used to compare the numerical variables. Logistic regression analysis was used to predict the dichotomous variables. The receiver operating characteristic curve and respective area under the curve were used to check the validity of the logistic regression models. Statistical significance was set at p-values less than 0.05. The statistical program R version 4.2 (R Foundation for Statistical Computing, Vienna, Austria) was used for statistical analysis.

## Results

**Figure 1:**
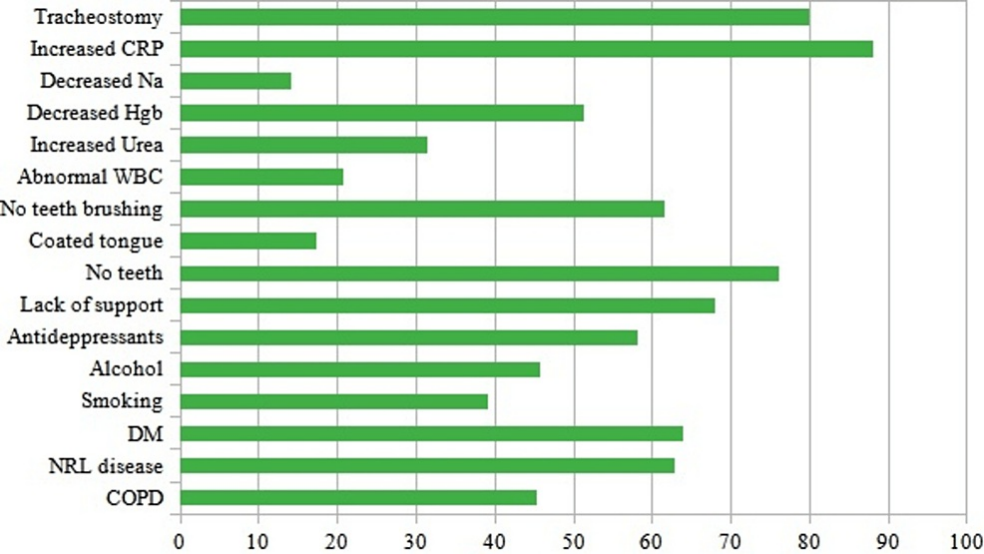
Prevalence of OD (in percentage %) in patients with CAP concerning other variables. The blood parameters were obtained at hospital discharge. OD: oropharyngeal dysphagia; CAP: community-acquired pneumonia; COPD: chronic obstructive pulmonary disease; NRL: neurological; DM: diabetes mellitus; WBC: white blood cells; Hgb: hemoglobin; Na: sodium; CRP: C-reactive protein.

Of the 452 patients recruited for the study, 226 had CAP, and 226 had a different acute respiratory diagnosis (control group), of whom 73 (31.4%) had COPD exacerbation. Comparative demographics and other variables are presented in Table 1. Patients with CAP were older, had more diabetes and neurological diseases, less COPD, took more antidepressants, had lower autonomy for basic activities of daily living, and had less institutional or familial support. In these patients, the complete absence of teeth was a frequent finding (Figure 1). However, the Charlson Comorbidity Index was not significantly different between groups (Table 1). The prevalence of pneumococcal and influenza vaccinations was low in both groups. OD was significantly more prevalent in patients with CAP than in the control group: 129 (57.1%) patients with CAP had a positive EAT-10 score, of whom 122 (94.5%) had an abnormal V-VST. In the control group, 46 (20.4%) patients had a positive EAT-10 score, of which 44 (95.6%) had an abnormal V-VST (p < 0.001; CI = 0.13-0.29). Impairments in both the safety and efficacy of swallowing were noted in most patients with CAP (87.7% and 90.2% had impaired safety and efficacy, respectively). In the CAP group, two variables were independently associated with OD in the multivariate analysis: age (p < 0.001; CI = 1.02-1.07) and institutional/familial support (p = 0.036; CI = 0.12-0.91). The prevalence of OD in the CAP group concerning other variables is shown in Figure 1.

**Table 1:** Patients with CAP and control group. Demographic data, comorbidities, and other variables in the two groups studied. CAP: community-acquired pneumonia; COPD: chronic obstructive pulmonary disease; N/A: non-applicable; NS: non-significant.

Variable	CAP	Control	p-value
Number of patients	226	226	NS
Sex: M (%), F (%)	M: 58.8, F: 41.4	M: 50, F: 50	p = 0.073
Age (years)	68.7 ± 15.9	64.8 ± 14.9	p = 0.008
BMI (kg/m^2^)	28.1 ± 5.5	28.0 ± 6.7	NS
Barthel scale score (0-100)	91.9 ± 20.2	97.9 ± 8.1	p < 0.001
CURB-65 scale on admission			
0, n (%)	38 (16.8)	N/A	
1, n (%)	39 (17.2)	N/A	
2, n (%)	118 (52.2)	N/A	
3, n (%)	28 (12.3)	N/A	
4, n (%)	3 (1.3)	N/A	
COPD, n (%)	48 (21.2)	73 (31.4)	p = 0.007
Asthma	26 (11.5)	17 (7.5)	NS
Neurological disease, n (%)	89 (39.3)	47 (20.7)	p < 0.001
Cardiovascular disease, n (%)	135 (59.7)	127 (56.1)	NS
Diabetes mellitus, n (%)	75 (33.1)	49 (21.6)	p = 0.008
Chronic renal failure, n (%)	33 (14.6)	33 (14.6)	NS
Gastrointestinal disease, n (%)	46 (20.4)	33 (14.6)	NS
Charlson Comorbidity Index, mean (SD)	3.32 (± 2.2)	3.16 (± 2.0)	NS
Use of benzodiazepines, n (%)	81 (35.8)	91 (40.3)	NS
Use of proton pump inhibitors, n (%)	145 (53.5)	126 (46.5)	NS
Use of antidepressants, n (%)	79 (35.0)	56 (24.8)	p = 0.024
Use of antipsychotics, n (%)	16 (7.1)	7 (3.1)	NS
Use of opioids, n (%)	28 (12.4)	28 (12.4)	NS
Pneumococcal vaccine, n (%)	7 (3.1)	3 (1.3)	NS
Flu vaccine, n (%)	36 (15.9)	5 (2.2)	p < 0.001
Current smoker, n (%)	56 (24.8)	60 (26.5)	NS
Ex-smoker, n (%)	83 (36.7)	79 (35.0)	NS
Current alcohol intake, n (%)	35 (15.5)	24 (10.6)	NS
Tracheostomy, n (%)	5 (2.2)	N/A	
Gastrostomy, n (%)	2 (0.8)	N/A	
Complete absence of teeth, n (%)	139 (61.5)	N/A	
Do not brush teeth, n (%)	151 (66.8)	N/A	
Institutional or family support, n (%)	201 (88.9)	224 (99.1)	p < 0.001
Predischarge analytical parameters			
Abnormal leucocytes, n (%)	25/122 (20.4)	N/A	
Increased urea, n (%)	38/122 (31.1)	N/A	
Decreased hemoglobin, n (%)	62/122 (50.8)	N/A	
Abnormal sodium, n (%)	17/122 (13.3)	N/A	
Increased C-reactive protein, n (%)	87/99 (87.8)	N/A	

Other results in the CAP group

**Figure 2:**
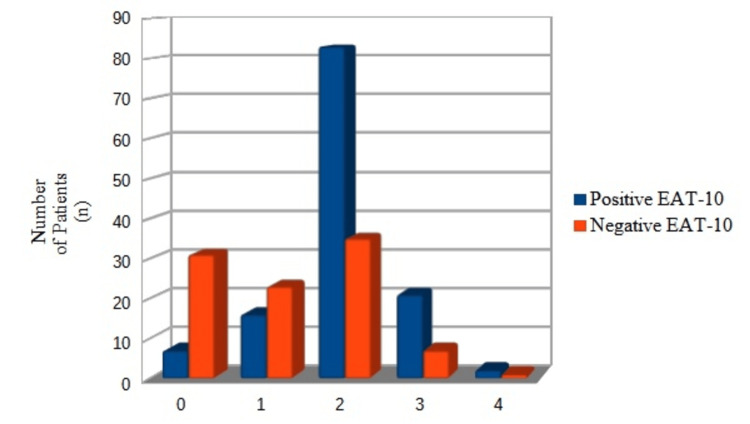
Distribution of patients with CAP according to the CURB-65 scale (0-4) and results of the EAT-10. CAP: community-acquired pneumonia; EAT-10: Eating Assessment Tool-10.

**Figure 3:**
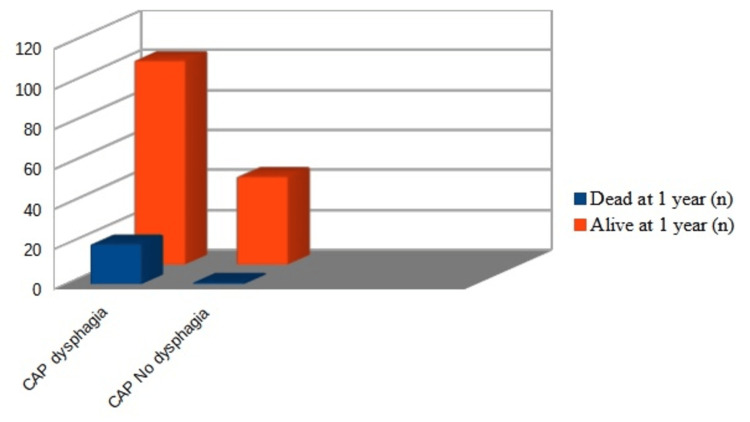
Vital status after one year in patients with CAP and OD compared with patients with CAP and no OD. See text for further details. OD: oropharyngeal dysphagia; CAP: community-acquired pneumonia.

The mean CURB-65 score in our series was 1.64 (+/- 0.95). According to this clinical prediction rule, the more severe the CAP, the higher the likelihood of OD (Figure 2). Those with a positive EAT-10 score had a lower mean Barthel score (88.5 points) than those with a normal EAT-10 (96.4 points) (p = 0.003). The mean hospital stay was 13.06 (+/- 13.2) days. The length of hospitalization was significantly longer in those with a positive EAT-10 score than in those with a negative score (14.5 (+/- 16.8) versus 11.0 (+/-5.3) days) (p = 0.038). However, OD was not found to be a determinant factor for the one-year readmission rate among patients with CAP: 18 (14.7%) patients with OD vs. 17 (16.3%) patients without OD, although it was associated with more visits to the ER (15 vs. 0). Of the 35 (15.4%) patients with CAP readmitted to the hospital during the follow-up, 12 had recurrent CAP, of whom nine had OD. Twenty (16.4%) patients with CAP and OD died during the following year after discharge vs. one (0.8%) patient with CAP and no OD (p < 0.001; CI = 2.24-42.60) (Figure 3).

## Discussion

The first and main finding of our study is that OD is frequent among hospitalized patients with CAP. This is in comparison to a large cohort of patients admitted to the respiratory ward for a diagnosis different than CAP but with similar weight of comorbidities. OD increases with age and is inversely related to institutional/familial support. Second, higher rates of OD were observed as the severity of CAP increased according to the CURB-65 prognostic scale. Third, in patients with CAP, OD was associated with prolonged hospital stays and more frequent visits to the ER after discharge. Among those readmitted with recurrent CAP, the prevalence of OD was high. Finally, we found that patients with CAP and OD had a significantly higher one-year death rate than those without OD. These findings are in agreement with those of other studies where OD was identified as a risk factor for pneumonia and mortality in patients with stroke and Down syndrome and was associated with multiple readmissions to the general wards and ICU [12,13]. According to Hägglund et al., the presence of OD increases the risk of pneumonia in the elderly, with an odds ratio of 11.9 [14], and poor functional status further increases the risk of OD [15]. Based on their results with the V-VST and videofluoroscopy, Almirall et al. recommended universal screening for OD in patients with CAP who are older than 70 years [16]. Other authors have reported that advanced age, poor functional status, chronic pulmonary disease, immunosuppression, inhaled corticosteroids, and lack of pneumococcal vaccination are risk factors for recurrent pneumonia; however, they did not focus on OD [4-6]. In terms of distinguishing CAP from aspiration pneumonia, the latter is estimated to account for 5-15% of CAP cases. Therefore, screening for OD may help identify patients at risk of aspiration [17]. Aspiration is often the result of impaired swallowing, which allows oral or gastric contents or both to enter the lung, especially if the cough reflex is impaired [18]. In an observational study of 1348 hospitalized patients with CAP, Taylor et al. reported that patients at risk for aspiration had a worse prognosis with increased mortality, a higher probability of readmission, and a strong association with recurrent admissions for pneumonia [19]. In our cohort of patients with CAP, the high prevalence of compromised swallowing safety and efficacy strongly suggests that aspiration pneumonia was underestimated.

Several studies have reported that early OD screening and subsequent intervention could reduce the incidence of pneumonia. Masuda et al. used videofluoroscopy to assess aspiration and penetration of fluids in the airway in ambulatory patients at risk for aspiration. They showed that thickening the liquids in the diet decreased aspiration and penetration, and consequently, reduced the risk of pneumonia [20]. In another study in the ICU, screening for OD helped determine the proper timing to initiate oral feeding in the first post-extubation days and was associated with a lower risk of recurrent pneumonia and length of hospital stay [20,21]. We have found a high prevalence of complete tooth loss and poor oral hygiene in patients with OD. Some studies have reported a relationship between tooth loss, caries, and periodontitis and an increased risk of death from pneumonia [22,23]. Improving oral hygiene and visiting the dentist may avert the progression or onset of respiratory diseases among high-risk older adults, while the increased frequency of oral care in the acute care setting may decrease the risk of hospital-acquired pneumonia [23,24].

Limitations

The specificity of V-VST might be suboptimal in certain cases, and videofluoroscopy or fiberoptic endoscopic evaluation of swallowing (FEES) could be considered after a positive V-VST [25]. However, a recent review of the available evidence of V-VST in the diagnosis of OD reported good sensitivity and specificity of the test (93% and 81%, respectively) [25,26]. This reinforces, in good hands, the validity of the procedure, which may suffice as a diagnostic method, especially when instrumental tests are not readily available. As stated, an important challenge lies in labeling pneumonia as CAP or aspiration pneumonia. However, the EAT-10 and V-VST results may assist in delivering recommendations and optimizing the outcomes. As mentioned, aspiration pneumonia has been associated with higher severity and worse prognosis. We found that OD was more prevalent among those with a higher CURB-65 score. Patients with aspiration pneumonia may have had more severe pneumonia, but this remains speculative as we cannot provide a clear association in this regard. The prevalence of OD is estimated to be 20% in patients with stable COPD. This prevalence may increase during exacerbations [27]. In our study, the presence of OD in the control group may have been overestimated because COPD was more frequent in this group. Despite this finding, OD was significantly less prevalent in the control group than in the CAP group. Regarding the timing of the test, Macht et al. found that among extubated patients with respiratory failure, the prevalence of OD persisted at hospital discharge in 35% of those initially identified with endoscopically confirmed aspiration [28]. By applying the V-VST immediately before discharge, we believe that we reduced the risk of false-positive results.

Studies focusing on preventive strategies for modifiable risk factors of CAP and aspiration pneumonia (screening OD with changes in diet, oropharynx colonization, and gastric pH) are required [29,30]. In cases of OD with a risk of aspiration, keeping the patient’s chin down and head turned to one side during feeding and encouraging swallowing of small volumes, multiple swallows, and coughing after each swallow should be encouraged. Modifications to the viscosity of dietary solids and liquids are necessary (e.g., by adding thickening powder to liquids), and rehabilitation of swallowing or even gastrostomy could be required in some circumstances (e.g., recurrent aspiration pneumonia despite compliance with appropriate diet) [15,30].

## Conclusions

We conclude that OD was almost three times more prevalent in patients with CAP than in those hospitalized for other respiratory diagnoses. The prevalence of dysphagia increases with the severity of pneumonia and is associated with older age, less familial/social support, longer hospital stays, more visits to the ER, and decreased survival rates in the year after diagnosis. The EAT-10 is an excellent predictor of OD in patients with CAP and other acute respiratory disorders, later confirmed by V-VST. In patients with CAP and OD, aspiration pneumonia is likely underestimated. Our study may help increase the awareness of OD as a risk factor in patients with CAP. Once OD is identified, interventions at different levels, e.g., diet, nutrition, oral hygiene, and rehabilitation, will most likely ameliorate these outcomes and would be cost-effective.
